# Gene Expression Analysis for Uterine Cervix and Corpus Cancer Characterization †

**DOI:** 10.3390/genes15030312

**Published:** 2024-02-28

**Authors:** Lucía Almorox, Laura Antequera, Ignacio Rojas, Luis Javier Herrera, Francisco M. Ortuño

**Affiliations:** Department of Computer Engineering, Automatics and Robotics, C.I.T.I.C., University of Granada, Periodista Rafael Gómez Montero, 2, 18014 Granada, Spain; luciaalmorox@correo.ugr.es (L.A.); lantequera@ugr.es (L.A.); jherrera@ugr.es (L.J.H.); fortuno@ugr.es (F.M.O.)

**Keywords:** uterine corpus cancer, cervical cancer, cervical adenocarcinoma, cervical squamous cell carcinoma, KnowSeq, RNA-Seq, MicroRNAs, differentially expressed genes, gene signature

## Abstract

The analysis of gene expression quantification data is a powerful and widely used approach in cancer research. This work provides new insights into the transcriptomic changes that occur in healthy uterine tissue compared to those in cancerous tissues and explores the differences associated with uterine cancer localizations and histological subtypes. To achieve this, RNA-Seq data from the TCGA database were preprocessed and analyzed using the KnowSeq package. Firstly, a kNN model was applied to classify uterine cervix cancer, uterine corpus cancer, and healthy uterine samples. Through variable selection, a three-gene signature was identified (*VWCE*, *CLDN15*, *ADCYAP1R1*), achieving consistent 100% test accuracy across 20 repetitions of a 5-fold cross-validation. A supplementary similar analysis using miRNA-Seq data from the same samples identified an optimal two-gene miRNA-coding signature potentially regulating the three-gene signature previously mentioned, which attained optimal classification performance with an 82% F1-macro score. Subsequently, a kNN model was implemented for the classification of cervical cancer samples into their two main histological subtypes (adenocarcinoma and squamous cell carcinoma). A uni-gene signature (*ICA1L*) was identified, achieving 100% test accuracy through 20 repetitions of a 5-fold cross-validation and externally validated through the CGCI program. Finally, an examination of six cervical adenosquamous carcinoma (mixed) samples revealed a pattern where the gene expression value in the mixed class aligned closer to the histological subtype with lower expression, prompting a reconsideration of the diagnosis for these mixed samples. In summary, this study provides valuable insights into the molecular mechanisms of uterine cervix and corpus cancers. The newly identified gene signatures demonstrate robust predictive capabilities, guiding future research in cancer diagnosis and treatment methodologies.

## 1. Introduction

Gynecological cancers present a significant global health concern, with an estimated annual incidence surpassing 3.6 million and a mortality rate exceeding 1.3 million. They contribute to nearly 40% of all cancer incidence and over 30% of cancer-related deaths in women worldwide [1]. Uterine cervix and uterine corpus (body) cancers rank as the top two gynecological malignancies globally. Despite their proximity within the female reproductive system, these cancers exhibit substantial differences in etiology, risk factors, and disease characteristics. Additionally, the impact of each uterine cancer varies depending on the socioeconomic conditions and lifestyle factors of each country [2]. In developing countries, cervical cancer constitutes the most prevalent gynecological malignancy [3]. The decrease in incidence and mortality observed in developed countries over the past few decades is largely attributed to highly effective prevention measures (screening tests and HPV vaccination programs), which, unfortunately, remain relatively limited in accessibility in developing nations [2]. Conversely, in developed countries, uterine corpus cancer stands as the most prevalent gynecological malignancy, surpassing cervical cancer [4,5]. This prevalence is notably associated with a specific risk factor for the disease: obesity, a condition more commonly observed in middle- and high-income countries [2].

Each uterine cancer type comprises various histological subtypes, each associated with distinct prognoses, responses to treatment, and risk factors. The primary histological subtypes of cervical cancer include squamous cell carcinoma (70–75%) [6], which develops from cells in the ectocervix [7], and adenocarcinoma (10–25%) [6], originating in the glandular cells of the endocervix [7]. In comparison to cervical squamous cell carcinoma, cervical adenocarcinoma exhibits greater aggressiveness, a higher rate of metastasis, inferior prognosis, and reduced rates of survival [8]. Cervical adenosquamous carcinoma is an infrequent subtype (with an incidence rate of less than 6/100,000 [9]) that is characterized by the presence of both squamous cell and glandular differentiation [10]. Few reports, often inconsistent, have documented the survival outcomes and prognostic factors in patients with this histological subtype [10].

Uterine corpus cancer is divided into two primary subtypes. Adenocarcinoma, which makes up the majority of uterine cancers, develops from cells in the endometrium or uterine lining and is commonly referred to as endometrial cancer. The second subtype is sarcoma (2–4%), which develops in the supporting tissues of the uterine glands or in the myometrium, the uterine muscle [11]. While endometrial cancer is frequently curable, uterine sarcoma is often more aggressive and harder to treat [12]. Uterine corpus carcinosarcomas are infrequent (<0.005%) mixed tumors that histologically comprise both epithelial and mesenchymal structures. Among the three histological subtypes, this is the one with the worst prognosis, as it is prone to metastasis and recurrence [13].

The heterogeneity within cancer underscores the need to deepen our understanding of the molecular pathogenesis of each cancer type, including histological subtypes. This exploration is essential for identifying new therapeutic targets and improving methods for precise diagnosis and personalized management strategies.

Differential expression analysis using high-throughput techniques on biological samples enables the characterization of normal cell physiology and the alterations occurring during cancer progression. This study utilizes this analytical framework to identify novel gene signatures for the classification of uterine cancers, thus contributing to their molecular characterization. To achieve this, RNA-Seq data and miRNA-Seq data from uterine cancer samples were obtained from the TCGA database, and a supervised machine learning approach, supported by the bioinformatics package KnowSeq [14], was implemented.

Firstly, a three-gene signature was established to differentiate between healthy uterine, cervical cancerous, and uterine corpus cancerous samples. Subsequently, a miRNA signature was determined for the same classification task, offering additional insights into molecular differences. Following tissue classification, the study addressed the distinction between primary histological subtypes of cervical cancer (adenocarcinoma and squamous cell carcinoma) and investigated the characterization of mixed tumors compared to the main histological subtypes.

This work extends the paper published at the IWBBIO 2023 conference [15] by renewing the experiments with updated datasets, conducting parallel miRNA-Seq analysis, and improving robust validation. The novelty of the results obtained from this study is summarized as follows: In the k-Nearest Neighbors (kNN) classification of healthy uterine, cervical cancerous, and uterine corpus cancerous samples, the original three-gene signature was refined by substituting the third gene, *SERTM1*, with *ADCYAP1R1*. This adjustment significantly improved the F1 value, increasing from 96.73% to a perfect 100% when utilizing the complete signature (*VWCE*, *CLDN15*, and *ADCYAP1R1*). Additionally, the miRNA-Seq analysis revealed a two-gene miRNA-coding signature (*hsa-mir-21* and *hsa-mir-10b*), achieving an F1-macro score of 82% in the same classification task. Concerning the kNN classification of primary histological subtypes of cervical cancers, the initial paper identified the gene *ICA1L* as one of the top DEGs between these subtypes. In this study, we evaluated its performance as a biomarker, establishing a single-gene signature. The results were optimal, and external validation for this gene signature was feasible using CGCI program samples. Finally, the examination of six cervical adenosquamous carcinoma (mixed) samples in relation to the two main histological subtypes of cervical cancer provided novel insights that can potentially influence the reconsideration of diagnosis for these mixed samples.

## 2. Materials and Methods

### 2.1. Classification of Healthy, Cervical Cancer, and Uterine Corpus Cancer Samples

#### 2.1.1. Data Collection and Preprocessing

The Genomic Data Commons (GDC) portal (https://portal.gdc.cancer.gov/, version 1.0, accessed on 25 December 2023) was used for downloading the data from The Cancer Genome Atlas (TCGA, https://www.cancer.gov/ccg/research/genome-120sequencing/tcga, accessed on 25 December 2023). Specifically, *corpus uteri* and *cervix uteri* were selected as primary sites as part of CESC, UCEC, and SARC TCGA projects. From that, all STAR-Counts files included as *Gene Expression Quantification* data types were downloaded. The associated sample sheet and clinical table were also retrieved and are accessible in the work’s GitHub repository.

Upon retrieval of the data from the GDC, preprocessing steps were undertaken to prepare for subsequent analyses. Initially, undersampling was applied to the cancerous classes to address the imbalance in the original dataset (refer to Table 1). Subsequently, utilizing the KnowSeq package [14,16] (version 3.18), the data underwent the following procedures: the transformation of count data into gene expression values for each sample through conditional quantile normalization [17], the identification and removal of outliers (i.e., samples exhibiting expression distributions markedly different from the rest), and the application of surrogate variable analysis (SVA) to mitigate batch effects [18]. Table 1 provides an overview of the number of samples of each class downloaded from the GDC, as well as the number remaining after undersampling and after outlier removal.

#### 2.1.2. Identification of the Best Feature Selection Method

To select the most appropriate feature selection method for our classification task, the three methods available in KnowSeq were evaluated: MRMR (maximum relevance, minimum redundancy) [19], RF (random forest as a feature selector) [14], and DA (disease association, which ranks genes based on their biological association with the disease of interest, in this case, uterine disease) [14]. For this purpose, the high-quality samples were randomly split into a training set and a test set, using an 80–20% scheme (see Table 2).

The training set was subjected to the KnowSeq *DEGsExtraction* function based on the limma library [20], which performed an analysis to extract DEGs in the three classes of interest. This function was configured with a significance level of 0.001, determined using the t-statistic test moderated by an empirical Bayes method [21], and adjusted using the Benjamini–Hochberg method (BH) [22] to control the false discovery rate. Note that since this is a multiclass problem, the t-statistic was applied pairwise to sample classes, avoiding the classical biclass pipeline, which limma implements by default. Parameters were set to a value of 2 for both *lfc* (minimum log2 fold change) and *cov* (minimum coverage). *Cov* is a parameter in KnowSeq that represents the pairs of distinct sample conditions between which a particular gene can differentiate [23]. To be considered a DEG, a gene must meet all three criteria (*p*-value, coverage, and logFC). The resulting DEGs expression matrix was subjected to KnowSeq *FeatureSelection* function, which was set to three distinct mode options (*mrmr*, *rf*, or *da*) to prioritize genes based on their significance in predicting the sample class. Afterward, for each ranking, the KnowSeq *knn_train* function was executed on the training sample set. This function normalized the data, optimized the value of the number of *k* neighbors, and trained 10 kNN models, utilizing the top 1 to 10 genes from the corresponding ranking as features. Subsequently, the KnowSeq *knn_test* function allowed the assessment of each model’s effectiveness on the test sample set. The original source code of this function was modified to perform min–max normalization on the training and test sets jointly.

#### 2.1.3. 5-Fold Cross-Validation Assessment Using MRMR as the Feature Selection Method—Gene Signature Identification

Based on the findings from the preceding experiment, MRMR emerged as the most effective method for feature selection, hence its employment for the subsequent phases of this work. To mitigate the potential impact of sampling variability, the next step involved evaluating the model’s performance across multiple training–test partitions of the dataset. In particular, a 5-fold cross-validation assessment process was performed. For the training set in each fold, DEGs were extracted, and an MRMR ranking of 10 genes was obtained. An increasing number of genes from the ranking was used to train 10 kNN models. For each number of genes, mean accuracies among the five folds were calculated. After inspecting the MRMR rankings from each fold, a reduced gene signature was proposed as the final feature selection for this classification task.

#### 2.1.4. 5-Fold Cross-Validation Assessment Using the Gene Signature as the Feature Selection

An additional 5-fold cross-validation was performed, where the same feature selection was used consistently across folds—namely, the genes comprising the signature identified in the prior cross-validation. To minimize the impact of the 5-fold partition on results, 20 iterations of the cross-validation were carried out, varying the random seed for the generation of pseudo-random numbers.

#### 2.1.5. Functional Annotation of the Gene Signature

Functional annotation of the genes was conducted manually utilizing resources such as GeneCards (https://www.genecards.org/, version 5.18, accessed on 3 January 2024) and the National Library of Medicine (https://www.ncbi.nlm.nih.gov/, accessed on 3 January 2024). Moreover, a comprehensive literature review was carried out to explore relationships between each gene and uterine cancer or, alternatively, cancer in general. In addition to manually searching for pertinent articles, the VarElect tool (https://ve.genecards.org/, accessed on 3 January 2024) was also utilized for the gene-phenotype association search.

#### 2.1.6. Complementary miRNA Sample Analysis

To carry out the study with miRNA samples, an analysis similar to the one described above for RNA-Seq was conducted over the same TCGA projects. To avoid extending the length of this work, a supplementary document is included (see Supplementary Materials), providing detailed information on the methodology and presenting the different results obtained. Only the most relevant results with miRNA data are briefly discussed in the main manuscript.

### 2.2. Classification of Cervical Adenocarcinoma and Cervical Squamous Cell Carcinoma Samples

After confirming an adequate representation of the primary histological subtypes of cervical cancer (adenocarcinoma and squamous cell carcinoma) within the downloaded GDC samples, we proceeded to train the kNN model for classifying cervical cancer samples into these two subtypes. To focus solely on cervical cancer, samples related to uterine corpus cancer or healthy tissue were excluded from the analysis.

The preprocessing of the raw cervix cancer samples followed a similar approach to the previous classification task. The primary distinction involved relabeling these samples (*ADENO*—48 samples or *SQUAMOUS*—251 samples) based on information extracted from the *primary_diagnosis* field in the clinical dataset (refer to Table 3). Five samples could not be classified into any of these classes because their histological subtype was “Adenosquamous carcinoma”. These five mixed samples were eliminated. After removing the outliers, there were 46 *ADENO* and 244 *SQUAMOUS* samples. The steps followed for the identification of a gene signature and its assessment for this classification task were analogous to those explained for the previous classification. In this scenario, KnowSeq utilized the limma biclass pipeline to extract DEGs.

#### External Validation of the Gene Signature for the Classification of the Main Histological Subtypes of Cervical Cancer

Through the GDC, we obtained additional cervical cancer samples from the Cancer Genome Characterization Initiative (CGCI) program, specifically from the HTMCP-CC project. Operating independently of TCGA, our goal was to externally validate the gene signature designed for classifying histological subtypes of cervical cancer. In this new GDC download, samples from both TCGA-CESC and CGCI-HTMCP-CC were acquired simultaneously (the sample sheet and associated clinical tables are accessible in the work’s GitHub repository). This facilitated the application of the preprocessing pipeline to the combined set of cervical samples from both projects, a crucial step to address potential batch effects that could lead to unexpected outcomes. Given that the CGCI-HTMCP-CC dataset had fewer samples than the TCGA-CESC project, the initial step involved undersampling the TCGA-CESC samples, specifically from the majority class, *SQUAMOUS*. The number of samples of each class from each project is displayed in Table 4. After obtaining the preprocessed combined dataset, samples from the TCGA-CESC project were utilized to train the kNN model with the gene signature previously obtained. Subsequently, samples from the CGCI-HTMCP-CC project were used to test this model.

### 2.3. Comparing the Adenosquamous Class with the Main Histological Subtypes of Cervical Cancer

The TCGA-CESC project included five adenosquamous (or mixed) cervix cancer samples, whereas the CGCI-HTMCP-CC project had one. As previously mentioned, these samples were excluded from the kNN classification of cervix cancer subtypes. Nevertheless, we conducted an additional experiment to explore the expression of the top 12 DEGs (genes with the lowest logFC among the two main histological subtypes) in these mixed samples. These DEGs were identified using the combined dataset from both TCGA-CESC and CGCI-HTMCP-CC samples, employing the KnowSeq *DEGsExtraction* function, as described in Section 2.1.2.

Note: The work’s GitHub repository contains all the R Markdown files used to create this article: https://github.com/Almorox/MDPI_Journal_GENES_Uterine_Cancers-Characterization_through_Gene_Expression_Analysis (accessed on 29 January 2024).

## 3. Results and Discussion

### 3.1. Classification of Healthy, Cervical Cancer, and Uterine Corpus Cancer Samples

#### 3.1.1. Identification of the Best Feature Selection Method

**Figure 1:**
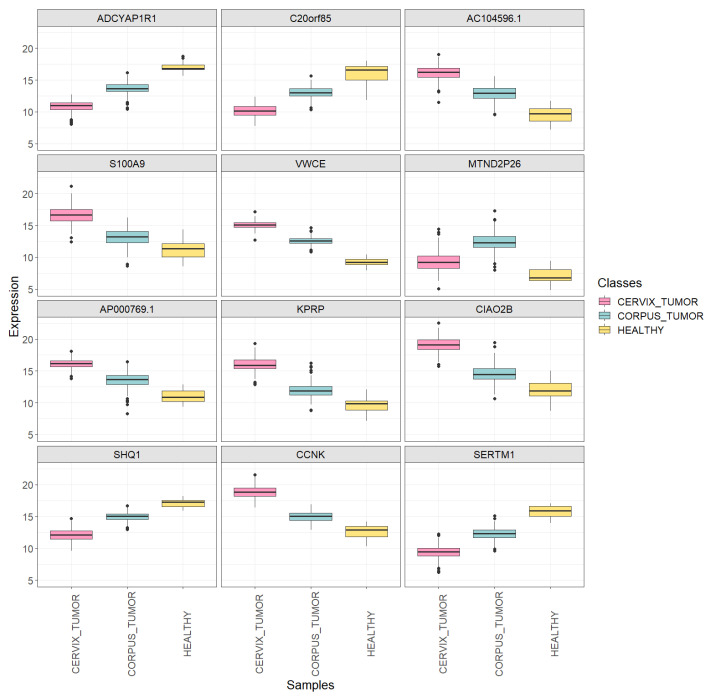
Boxplots showing the expression of the first 12 extracted DEGs in each uterine sample class (*CORPUS_TUMOR*, *CERVIX_TUMOR* and *HEALTHY*) using the training set.

Using the training set (Table 2) and the specified statistical parameters, 19 genes were detected as DEGs across the three classes. Examining the first 12 extracted DEGs, it can be observed that, for most of them (with the exception of the *MTND2P26* gene), the average expression level in uterine corpus cancer samples lay between those of the other two sample classes (see Figure 1, with associated adjusted *p*-values in Table 5). This indicates that the classifier is expected to successfully differentiate between cervical cancer and healthy classes but may encounter challenges in distinguishing uterine corpus cancer from either of the other two classes.

**Figure 2:**
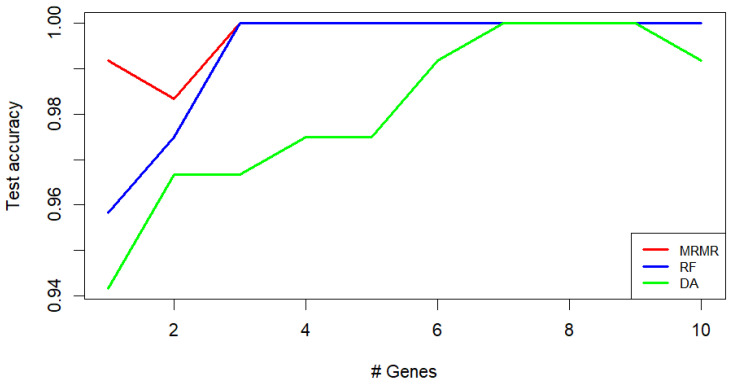
Classification of *CORPUS_TUMOR*, *CERVIX_TUMOR*, and *HEALTHY* uterine samples: kNN test accuracy obtained using different feature selection methods (MRMR, RF, and DA). The values are presented as a function of the number of genes used.

Remarkably, the MRMR and RF feature selection methods achieved a kNN test accuracy of 1 when three genes were considered (see Figure 2). However, when employing a smaller number of genes, the MRMR method appears to be more effective. Therefore, MRMR was chosen as the feature selection method for this work.

#### 3.1.2. 5-Fold Cross-Validation Using MRMR as the Feature Selection Method

**Figure 3:**
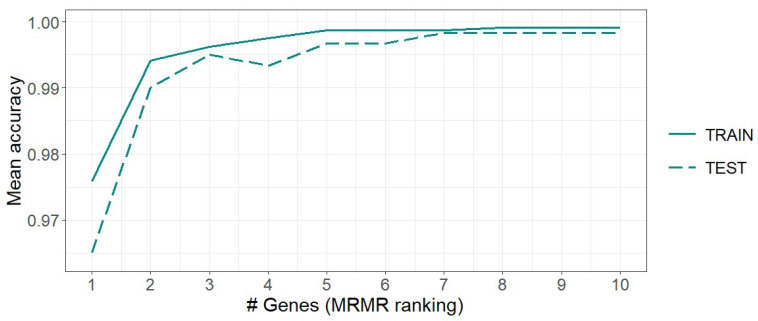
Classification of *CORPUS_TUMOR*, *CERVIX_TUMOR*, and *HEALTHY* uterine samples: kNN mean training and testing accuracy of the 5-fold cross-validation using MRMR. The values are presented as a function of the number of genes used.

Figure 3 illustrates that by utilizing only two MRMR-selected genes, the average test accuracy across folds reaches 0.99, suggesting a promising outcome.

**Figure 4:**
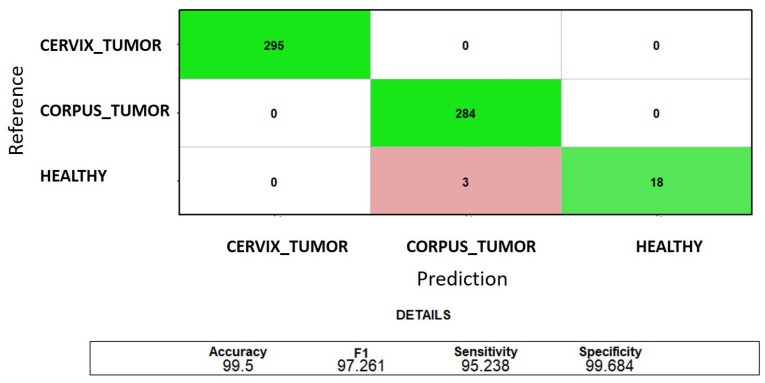
Classification of *CORPUS_TUMOR*, *CERVIX_TUMOR*, and *HEALTHY* uterine samples: the sum of the test confusion matrices of each fold of the 5-fold cross-validation when using the first three MRMR-selected genes. The green color indicates correct predictions, and the red color indicates incorrect predictions.

Nevertheless, it is crucial to recognize that the healthy class is severely underrepresented, meaning that high error rates in its classification have minimal impact on the overall classification accuracy. This is why another insightful graphical representation of the classification results is the sum of the test confusion matrices from each fold. This matrix offers a comprehensive overview of the successes and failures of the classification process across all quality samples. In particular, as depicted in Figure 4, when utilizing three genes, the sole type of error observed is the misclassification of healthy samples as samples of uterine corpus cancer, with an error rate of 14.28%. The occurrence of the highest error rate in the classification of the least represented class contributes to a decline in the overall test F1 value (97.26%) compared to that of precision (99.5%).

Among the top two MRMR-selected genes from the 5-fold cross-validation, only the *VWCE* and *CLDN15* combination is observed in more than one fold (see Table 6). In fold 1, this combination is followed by the *ADCYAP1R1* gene, whereas in fold 4, it is followed by *SERTM1*. Based on Table 5, *ADCYAP1R1* exhibits lower adjusted *p*-values for differential expression compared to *SERTM1* across all three pairs of sample classes. Therefore, the genes *VWCE*, *CLDN15*, and *ADCYAP1R1* were selected to compose a gene signature, with which the 5-fold cross-validation was repeated.

#### 3.1.3. 5-Fold Cross-Validation Using the Gene Signature for Feature Selection

**Figure 5:**
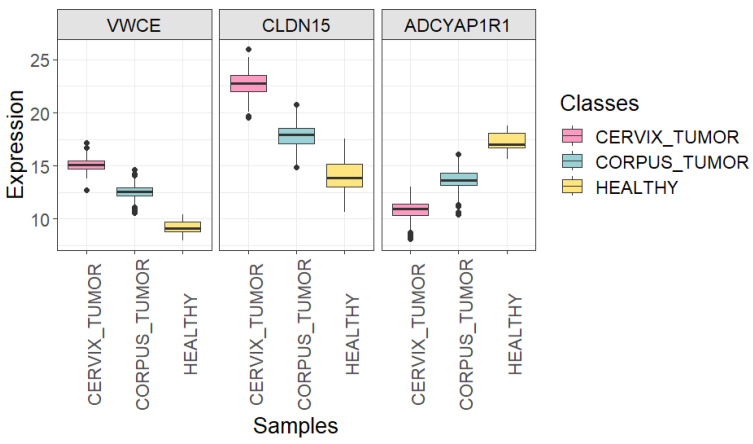
Boxplots showing the expression of *VWCE*, *CLDN15*, and *ADCYAP1R1* genes in each uterine sample class (*CORPUS_TUMOR*, *CERVIX_TUMOR*, and *HEALTHY*) using all quality samples.

**Figure 6:**
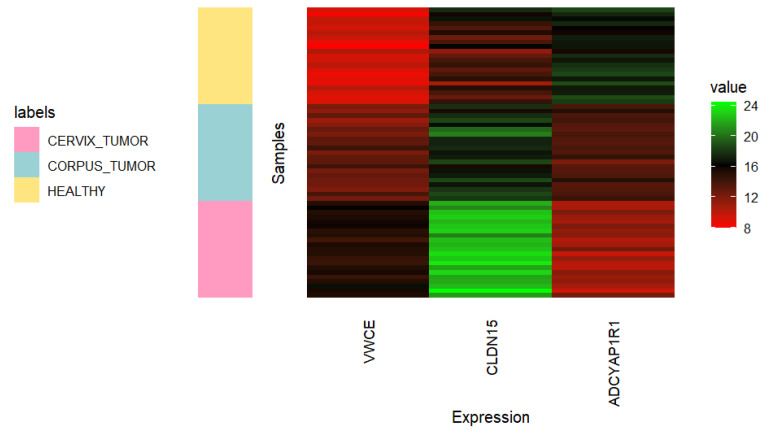
Heatmap showing the expression of *VWCE*, *CLDN15*, and *ADCYAP1R1* genes in 21 randomly selected samples of each uterine class (*CORPUS_TUMOR*, *CERVIX_TUMOR*, and *HEALTHY*). Undersampling of cancerous classes was carried out using all quality samples.

Figure 5 (with associated adjusted *p*-values in Table 7) and Figure 6 allow us to gain insight into the ability of *VWCE*, *CLDN15*, and *ADCYAP1R1* genes to effectively distinguish between the three classes of interest. The genes *VWCE* and *CLDN15* exhibit overexpression in cancerous uterine tissue compared to healthy uterine tissue. However, this overexpression is more pronounced in the cervix than in the corpus. Conversely, the gene *ADCYAP1R1* demonstrates underexpression in cancerous uterine tissue compared to its healthy counterpart, with this underexpression being more accentuated in the cervix than in the corpus.

**Figure 7:**
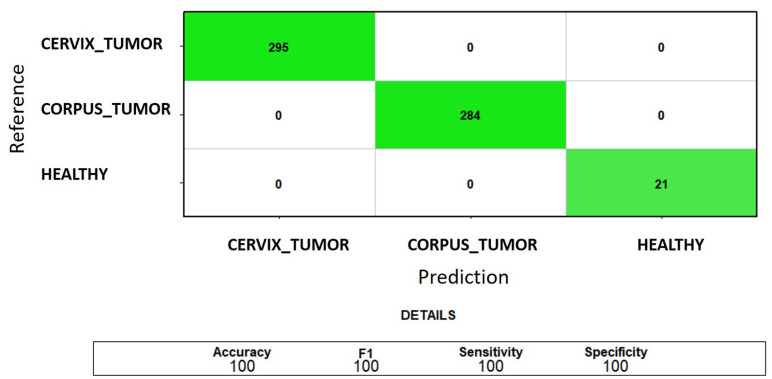
Classification of *CORPUS_TUMOR*, *CERVIX_TUMOR*, and *HEALTHY* uterine samples: sum of the test confusion matrices of each fold of the 5-fold cross-validation using the complete gene signature (*VWCE*, *CLDN15*, and *ADCYAP1R1*) for feature selection. The green color indicates correct predictions.

The mean training and test accuracy values achieved through 5-fold cross-validation using *VWCE*, *CLDN15*, and *ADCYAP1R1* as selected features were 100%. This optimal result remained constant across the 20 iterations of the 5-fold cross-validation (carried out by varying the seed). Hence, the gene signature perfectly characterizes the three sample classes (see Figure 7) and corrects the misclassification of healthy samples as uterine corpus cancerous observed when using alternative MRMR-selected genes.

#### 3.1.4. Gene Signature Annotation

*VWCE* (Von Willebrand Factor C And EGF Domains) Gene. Biological functions attributed to *VWCE* (also referred to as *URG11*) include its predicted involvement in calcium ion binding activity, cellular response to viruses, and localization within the cytoplasm. It may also serve as a regulatory component in the beta-catenin signaling pathway and be a potential target for the chemoprevention of hepatocellular carcinoma. Diseases linked to *VWCE* include tarsal-carpal coalition syndrome [24]. Furthermore, there exist studies associating this gene with diverse types of neoplasms [25]. For instance, it was found to be downregulated in breast and prostate cancers, where it functions as a tumor suppressor [26,27]. Interestingly, another project report mentioned the observation of *VWCE* overexpression in cervical cancer [28], which aligns with our findings. However, the role of *VWCE* gene in uterine cancers remains unclear.

*CLDN15* (Claudin 15) Gene. This gene is responsible for encoding a member of the claudin family. Claudins play a crucial role in forming tight junction strands, which act as a barrier to regulate the movement of solutes and water across the paracellular space between epithelial or endothelial cell layers. *CLDN15* is involved in pathways such as the blood–brain barrier and immune cell transmigration, with its associated diseases, including collagenous colitis [24]. It was reported as a good positive marker for malignant pleural mesothelioma [29]. While alterations in the expression of other claudins in uterine cancers have been documented, with notable increases in claudins-1 and -7 observed in premalignant cervical lesions and invasive cancer compared to normal cervical epithelia, as well as elevated levels of claudins-3 and -4 in endometrial cancer [30], the role of *CLDN15* in uterine cancers remains largely undescribed.

*ADCYAP1R1* (ADCYAP Receptor Type I) Gene. This gene encodes a membrane-associated receptor for ADCYAP1 (adenylate cyclase-activating polypeptide 1), mediating various biological actions of this ligand. Specifically, it may regulate the release of adrenocorticotropin, luteinizing hormone, growth hormone, prolactin, epinephrine, and catecholamine. It causes smooth muscle relaxation and secretion in the gastrointestinal tract. *ADCYAP1R1*-related pathways include signaling by NTRKs, and among its related diseases are accommodative spasm and sudden infant death syndrome. A study conducted by Jung et al. (2011) [31] detected a correlation between the level of *ADCYAP1* promoter hypermethylation (causing transcriptional silencing of this gene) and the development of cervical cancer. Our results can complement those of these authors, as we are identifying another factor causing the loss of ADCYAP1 functions in cervical cancer cells: the underexpression of one of its receptors.

#### 3.1.5. Complementary miRNA-Seq Analysis

A double miRNA-Seq analysis was performed on the miRNA data from the same samples. First, the whole miRNA dataset was employed, and MRMR revealed a gene signature reaching an F1-macro score of 82% with only four biomarkers. Then, the DIANA-TarBase v8 database [32] was used to recover the miRNAs targeting any of the genes in the RNA-Seq signature, and the same MRMR algorithm attained similar results with this small subset of miRNA. From both experiments, two miRNA-coding genes (*hsa-mir-21* and *hsa-mir-10b*) were identified as a double-gene miRNA-coding signature, demonstrating a mean F1-macro score of 82% by themselves during a 5-fold cross-validation assessment in the classification of the three types of uterine samples (cervix cancerous, corpus cancerous, and healthy). Specifically, *hsa-mir-21* is documented as a regulator of *VWCE*, and *hsa-mir-10b* as a regulator of *ADCYAP1R1*, potentially reinforcing the involvement of these protein-coding genes in the disease. Moreover, according to Sheedy et al. (2018) [33] and Rhim et al. (2022) [34], research findings indicate that both *hsa-mir-21* and *hsa-mir-10b* can significantly contribute to the promotion of tumor growth, invasion, and metastasis in various cancer types. Specifically, a study conducted by Huang et al. (2012) [35] revealed that *hsa-mir-10b* exhibited downregulation in advanced-stage of small cell cervical cancer (SCCC) tissues when compared to early-stage SCCC tissues.

### 3.2. Classification of Cervical Adenocarcinoma and Cervical Squamous Cell Carcinoma Samples

#### 3.2.1. 5-Fold Cross-Validation Using MRMR as the Feature Selection Method

The outcomes of the 5-fold cross-validation applied to classify cervical cancer samples into their primary histological subtypes (*ADENO* or *SQUAMOUS*) utilizing MRMR as the feature selection technique yielded optimal results. Regardless of the number of genes used (ranging from 1 to 10), both training and test accuracy values remained consistently at 100% across all folds. This underscores the significant discriminative potential of the genes identified by MRMR in distinguishing between these cancer subtypes. Furthermore, depending on the fold, the number of extracted DEGs varied between 1000 and 1005.

In this instance, the top one MRMR gene varies in each fold (see Table 8), which is likely a consequence of the composition of each fold’s training set. However, the gene in the second position remains constant: *ICA1L*. Therefore, we aimed to assess the performance of *ICA1L* as the sole final selected feature for this classification task, forming a uni-gene signature.

#### 3.2.2. 5-Fold Cross-Validation Using the Gene Signature for Feature Selection

The mean training and test accuracy values achieved through 5-fold cross-validation using *ICA1L* as the only selected feature were 100%. This optimal result remained constant across the 20 iterations of the 5-fold cross-validation, suggesting that the expression of the *ICA1L* gene alone is indicative enough to determine whether a cancerous cervix sample belongs to the adenocarcinoma or squamous cell carcinoma class.

**Figure 8:**
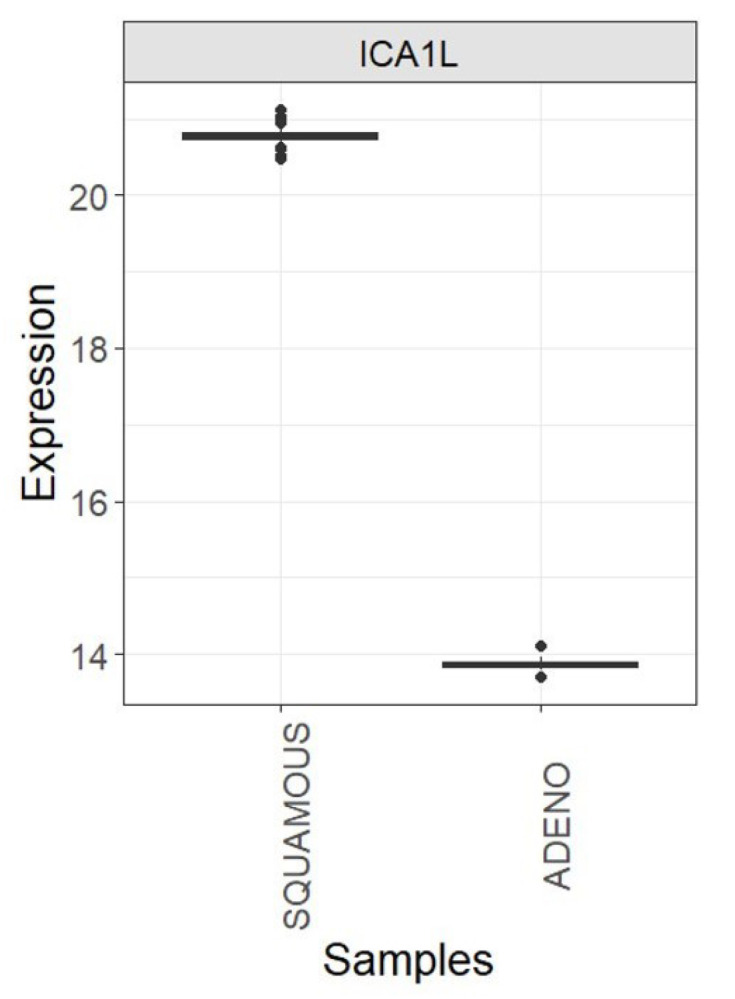
Boxplots showing the expression of the *ICA1L* gene in each cervix cancer class (*SQUAMOUS* and *ADENO*) using all TCGA-CESC quality samples.

Using all cervix cancer samples, the *ICA1L* differential expression among the two classes of interest was associated with a logFC of 6.92 and an adjusted *p*-value of 0, which explains the perfect kNN prediction. The clear distinction between the two sample classes is evident in the pronounced separation of their mean *ICA1L* expression values (see Figure 8). The *SQUAMOUS* class exhibits the highest value, and there is no overlap among the outlier values of each class.

#### 3.2.3. External Validation of the Uni-Gene Signature for the Classification of Histological Subtypes of Cervical Cancer

**Figure 9:**
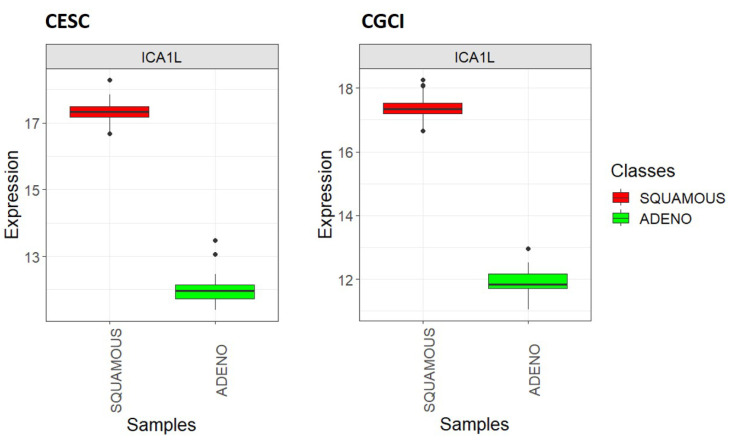
Boxplots showing the expression of the *ICA1L* gene in each cervix cancer class (*SQUAMOUS* and *ADENO*): TCGA-CESC vs. CGCI-HTMCP-CC samples after joint normalization of both datasets.

When using the TCGA-CESC sample set (original dataset) to train the kNN classifier and the CGCI-HTMCP-CC sample set (external dataset) to test the classifier, utilizing only the *ICA1L* gene as the selected feature, both the training and test accuracies were 100%. In this way, the utility of the *ICA1L* gene to distinguish the two main histological subtypes of cervix cancer was externally validated. Through Figure 9, it can be observed that the expression of *ICA1L* gene in the two classes of interest exhibits similar behavior in both datasets. This *ICA1L* differential expression was associated with a logFC of 5.36 and an adjusted *p*-value of 2.22 × 10^−178^ in the TCGA-CESC dataset, while these same parameters were 5.38 and 1.80 × 10^−129^, respectively, for the CGCI-HTMCP-CC dataset.

#### 3.2.4. Gene Signature Annotation

*ICA1L* (Islet Cell Autoantigen 1 Like) gene. This gene’s products are predicted to enable protein domain-specific binding activity, be involved in the regulation of transport, act upstream of or within spermatid development, be located in acrosomal vesicles, and be active in the Golgi apparatus. Diseases associated with *ICA1L* include amyotrophic lateral sclerosis type 2 (juvenile) and myofibroma [24]. It has been proposed that tumors with *SRF-ICA1L* fusions represent neoplasms exhibiting incomplete smooth muscle differentiation [36]. Nevertheless, the role of *ICA1L* in cervical cancer has not been described.

### 3.3. Comparing the Adenosquamous Class with the Main Histological Subtypes of Cervical Cancer

The top 12 DEGs, identified with the lowest logFC values, in the two major histological subtypes of cervical cancer using the combined dataset encompassing TCGA-CESC and CGCI-HTMCP-CC samples were *DSG3, MUC5B, MSMO1, AC026725.1, ICA1L, DSC3, AP1G1, CCDC89, SNED1-AS1, AC244034.3, AL360182.1*, and *FBXL13*. Refer to Table 9 to observe the logFC and adjusted *p*-values associated with each gene’s differential expression.

**Figure 10:**
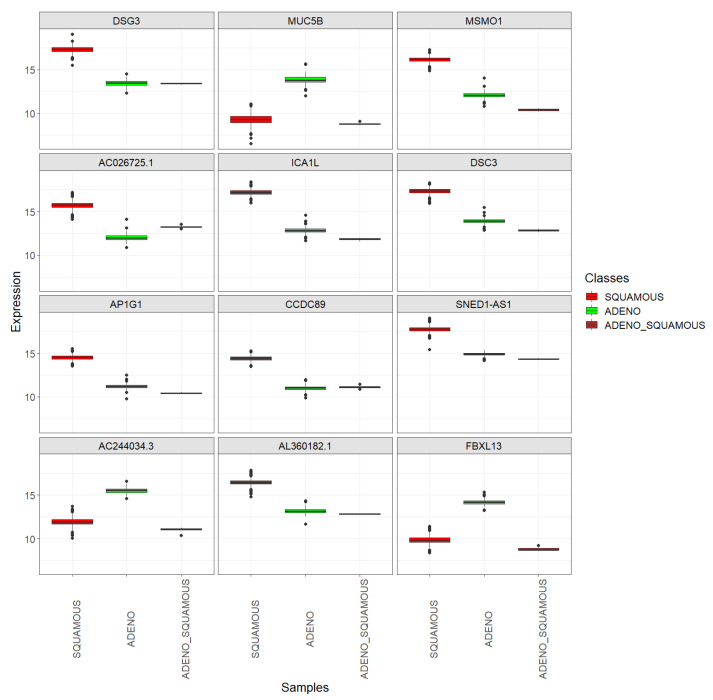
Top 12 DEGs between the *SQUAMOUS* and *ADENO* cervix cancer classes: boxplots showing their expression in the *SQUAMOUS*, *ADENO*, and *ADENO_SQUAMOUS* classes, using the combined dataset encompassing TCGA-CESC and CGCI-HTMCP-CC samples.

**Figure 11:**
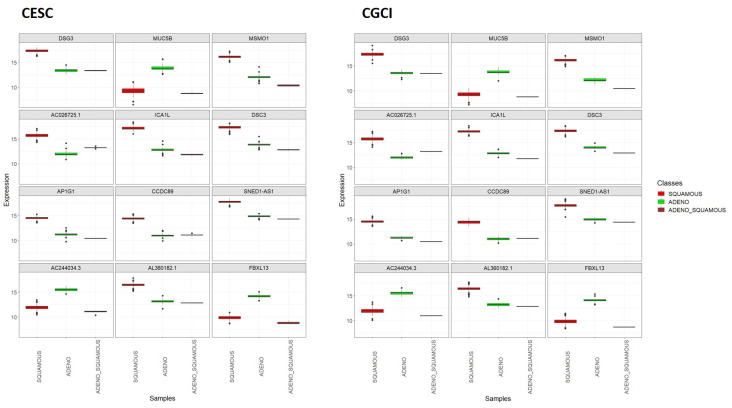
Top 12 DEGs between the *SQUAMOUS* and *ADENO* cervix cancer classes: boxplots showing their expression in the *SQUAMOUS*, *ADENO*, and *ADENO_SQUAMOUS* classes. TCGA-CESC vs. CGCI-HTMCP-CC samples after joint normalization of both datasets.

Figure 10 allows the observation of the expression of these genes in samples corresponding to the mixed subtype (adenosquamous carcinoma) and facilitates comparison with their expression in the two main subtypes. The same plot was constructed for TCGA-CESC and CGCI-HTMCP-CC samples separately (see Figure 11). This enables confirming that the expression values specific to each histological subtype are similar in both projects. It can be observed that the six adenosquamous samples have similar expression values with low dispersion. For nine of these genes (*DSG3, MSMO1, AC026725.1, ICA1L, DSC3, AP1G1, CCDC89, SNED1-AS1*, and *AL360182.1*), the mean expression value in the mixed class is closer to that of the *ADENO* class, while for the remaining three genes (*MUC5B, AC244034.3* and *FBXL13*), it is closer to that of the *SQUAMOUS* class. What is evident for the 12 genes is that the expression value in the mixed class is closer to that of the class in which the gene expression is lower, suggesting a lesser cellular differentiation in mixed tumors compared to the main subtypes.

Dedifferentiation is one manifestation of tumor plasticity, wherein cancerous cells lose their specialized characteristics and adopt less-differentiated phenotypes resembling those observed during early embryonic development or regenerative processes. The loss of differentiation is linked to heightened tumor cell invasiveness and resistance to drugs. Moreover, emerging evidence suggests its association with immune surveillance as well [37]. In squamous cell carcinoma, for instance, dedifferentiated tumor cells acquire stem-like properties and express the immune-modulating molecule CD80, enabling them to evade immune attacks [38].

Both the 2014 and 2020 World Health Organization (WHO) criteria require unequivocal glandular and squamous differentiation for diagnosing cervical adenosquamous carcinoma. Nevertheless, according to Stolnicu et al., in practice, diagnoses of this subtype are frequently made erroneously in tumors lacking unequivocal squamous and/or glandular differentiation [39]. A recent analysis of the morphology of this subtype revealed that, in 42% of cases initially diagnosed as adenosquamous carcinoma, a reclassification was necessary [9]. Thus, the observed pattern in our results, instead of providing insights for the characterization of cervical adenosquamous carcinoma, may suggest the need to reconsider the diagnosis of these six mixed samples, which appear to resemble dedifferentiated cervical tumors. Further investigation with a larger sample size is crucial to validate and extend these findings.

## 4. Conclusions and Future Work

The real gene expression quantification data obtained from TCGA underwent analysis using KnowSeq, enabling the identification of differentially expressed genes among healthy uterine tissue, cervical cancerous tissue, and uterine corpus cancerous tissue. Through the utilization of the MRMR feature selection method, a gene signature consisting of only three genes (*VWCE*, *CLDN15*, and *ADCYAP1R1*) was identified. The 5-fold cross-validation results were optimal, with overall training and test accuracies of 100%. This result remained consistent across 20 repetitions of the cross-validation. The three genes are protein-coding genes. *VWCE* and *CLDN15* have previously been associated with various neoplasms; however, their role in uterine cancers remains largely undescribed. In the case of ADCYAP1R1, its ligand (ADCYAP1) has been previously reported as transcriptionally silenced in cervical cancer. Hence, the observed underexpression of *ADCYP1R1* in this sample class may indicate an alternative mechanism for the inhibition of ADCYAP1 functions in cervical cancer cells. Moreover, concurrently with the miRNA-Seq study, a two-gene miRNA-coding signature (*hsa-mir-21* and *hsa-mir-10b*) was identified, exhibiting a remarkable 82% test F1-macro score for the same classification task. The DIANA-Tarbase documents these miRNAs as regulators of *VWCE* and *ADCYAP1R1*, respectively, potentially reinforcing the involvement of these genes in uterine cancer.

In a parallel experiment, cervical cancer samples were classified into their two primary histological subtypes: squamous cell carcinoma and adenocarcinoma. A uni-gene signature, defined by the *ICA1L* gene, achieved 100% training and test accuracies in a 5-fold cross-validation process. Once again, this result remained consistent across 20 repetitions of the cross-validation. External validation of this gene signature was possible through the CGCI-HTMCP-CC project. While *ICA1L* gene has previously been linked to neoplasms exhibiting incomplete smooth muscle differentiation, this gene’s role in cervical cancer has not been described.

Finally, the study examined the expression of 12 DEGs, identified between the main histological subtypes of cervical cancer, in six cervical adenosquamous carcinoma (mixed) samples. For all these genes, the expression value in the mixed class was closer to that of the class with lower gene expression, suggesting lesser cellular differentiation in mixed tumors compared to the main subtypes. This outcome may indicate the need for reconsidering the diagnosis of the six studied mixed samples.

Overall, this study offers valuable insights into the molecular mechanisms of cervical and uterine corpus cancers, setting the stage for future investigations aimed at enhancing diagnostic and treatment strategies. Subsequent research can delve into the presence and expression of HPV oncogenes in cervical cancer samples, contrasting them with samples from uterine corpus cancer and healthy uterine tissues. Moreover, exploring whether the identified gene signatures possess prognostic value by examining their associations with overall patient survival will be an intriguing approach for further exploration.

## Figures and Tables

**Table 1:** Downloaded, randomly selected (through undersampling), and filtered samples of each class.

Class	Description	Project	Downloaded	Rand. Selected	Quality Samples
CERVIX_TUMOR	Cervix cancer	TCGA-CESC	304	300	295
CORPUS_TUMOR	Uterine corpus cancer	TCGA-UCEC/SARC	552	300	284
HEALTHY	Non-cancerous cervix or uterine corpus	TCGA-CESC/UCEC/SARC	25	25	21

**Table 2:** Number of samples of each class in training and test sets.

Class	Training Samples	Test Samples
CERVIX_TUMOR	233	62
CORPUS_TUMOR	230	54
HEALTHY	17	4

**Table 3:** Number of downloaded cervical cancer samples that share the same primary diagnosis. All samples belonging to the first four subtypes in the table were labeled as *ADENO*; those belonging to the next 5 types as *SQUAMOUS* and those belonging to the last type were eliminated.

Histological Cervical Cancer Subtype	Downloaded Samples
Adenocarcinoma, endocervical type	21
Adenocarcinoma, NOS	7
Endometrioid adenocarcinoma, NOS	3
Mucinous adenocarcinoma, endocervical type	17
Squamous cell carcinoma, NOS	169
Papillary squamous cell carcinoma	1
Squamous cell carcinoma, keratinizing, NOS	30
Basaloid squamous cell carcinoma	1
Squamous cell carcinoma, large cell, nonkeratinizing	50
Adenosquamous carcinoma	5

**Table 4:** Cancer cervix samples from TCGA-CESC (original dataset) and CGCI-HTMCP-CC (external dataset) projects. Downloaded, randomly selected (through undersampling), and filtered samples of each class.

-	Class	Downloaded	Unders.	Quality Samples
CESC	ADENO	48	48	47
	SQUAMOUS	251	150	149
CGCI	ADENO	16	16	16
	SQUAMOUS	174	174	170

**Table 5:** Adjusted *p*-values (calculated by the t-statistic test moderated by an empirical Bayes method and adjusted using the BH method) for the differential expression of the first 12 extracted DEGs across each pair of uterine sample classes, using the training set.

Gene	*CERVIX_TUMOR-CORPUS_TUMOR*	*CERVIX_TUMOR-HEALTHY*	*CORPUS_TUMOR-HEALTHY*
*ADCYAP1R1*	$3.69\times 10^{-123}$	$5.04\times 10^{-98}$	$1.28\times 10^{-41}$
*C20orf85*	$1.11\times 10^{-119}$	$1.69\times 10^{-85}$	$2.00\times 10^{-31}$
*AC104596.1*	$9.71\times 10^{-116}$	$4.81\times 10^{-81}$	$6.27\times 10^{-29}$
*S100A9*	$6.86\times 10^{-100}$	$6.34\times 10^{-47}$	$4.38\times 10^{-9}$
*VWCE*	$6.19\times 10^{-173}$	$5.33\times 10^{-149}$	$9.19\times 10^{-75}$
*MTND2P26*	$3.03\times 10^{-76}$	$1.54\times 10^{-9}$	$2.16\times 10^{-39}$
*AP000769.1*	$5.78\times 10^{-101}$	$7.13\times 10^{-67}$	$4.44\times 10^{-22}$
*KPRP*	$8.42\times 10^{-150}$	$1.27\times 10^{-76}$	$5.68\times 10^{-15}$
*CIAO2B*	$6.04\times 10^{-155}$	$1.64\times 10^{-76}$	$1.84\times 10^{-13}$
*SHQ1*	$6.79\times 10^{-154}$	$3.92\times 10^{-91}$	$2.58\times 10^{-24}$
*CCNK*	$5.37\times 10^{-180}$	$8.43\times 10^{-104}$	$4.38\times 10^{-25}$
*SERTM1*	$3.43\times 10^{-119}$	$2.44\times 10^{-93}$	$1.15\times 10^{-38}$

**Table 6:** Classification of *CORPUS_TUMOR*, *CERVIX_TUMOR* and *HEALTHY* uterine samples: top 10 MRMR-selected genes for each fold (train set) of the 5-fold cross-validation.

-	Gene 1	Gene 2	Gene 3	Gene 4	Gene 5	Gene 6	Gene 7	Gene 8	Gene 9	Gene 10
Fold 1	*VWCE*	*CLDN15*	*ADCYAP1R1*	*CCNK*	*KCNK10*	*SHQ1*	*SERTM1*	*CCDC153*	*CD34*	*GDF11*
Fold 2	*CCNK*	*VWCE*	*SHQ1*	*GDF11*	*SERTM1*	*CLDN15*	*ADCYAP1R1*	*CCDC153*	*CD34*	*CPOX*
Fold 3	*S100A7*	*CD34*	*ADCYAP1R1*	*CCNK*	*VWCE*	*SHQ1*	*SERTM1*	*KCNK10*	*CLDN15*	*CPOX*
Fold 4	*VWCE*	*CLDN15*	*SERTM1*	*SHQ1*	*CCNK*	*ADCYAP1R1*	*KCNK10*	*CD34*	*CPOX*	*GDF11*
Fold 5	*VWCE*	*GDF11*	*CCNK*	*ADCYAP1R1*	*SHQ1*	*CLDN15*	*SERTM1*	*CPOX*	*CD34*	*SERPINB5*

**Table 7:** Adjusted *p*-values (calculated by the t-statistic test moderated by an empirical Bayes method and adjusted using the BH method) for the differential expression of *VWCE*, *CLDN15*, and *ADCYAP1R1* genes across each pair of uterine sample classes, using all quality samples.

Gene	*CERVIX_TUMOR-CORPUS_TUMOR*	*CERVIX_TUMOR-HEALTHY*	*CORPUS_TUMOR-HEALTHY*
*VWCE*	$8.35\times 10^{-217}$	$5.48\times 10^{-185}$	$5.89\times 10^{-92}$
*CLDN15*	$6.98\times 10^{-228}$	$2.89\times 10^{-144}$	$2.73\times 10^{-43}$
*ADCYAP1R1*	$3.88\times 10^{-157}$	$1.36\times 10^{-127}$	$8.09\times 10^{-56}$

**Table 8:** Classification of *SQUAMOUS* and *ADENO* cervix cancer samples: Top 10 MRMR-selected genes for each fold (training set) of the 5-fold cross-validation.

-	Gene 1	Gene 2	Gene 3	Gene 4	Gene 5	Gene 6	Gene 7	Gene 8	Gene 9	Gene 10
Fold 1	*DYRK3*	*ICA1L*	*GPR171*	*THPO*	*AC091563.1*	*RHOV*	*ZNF175*	*TINCR*	*ANXA8L1*	*POPDC3*
Fold 2	*ZNF812P*	*ICA1L*	*ZNHIT1*	*GPR171*	*TINCR*	*THPO*	*AC091563.1*	*RHOV*	*EPCAM*	*ZNF175*
Fold 3	*GABRQ*	*ICA1L*	*GPR171*	*THPO*	*AC091563.1*	*RHOV*	*ZNF175*	*SIPA1L3*	*CARD16*	*AC112907.2*
Fold 4	*SERPINB13*	*ICA1L*	*GPR171*	*THPO*	*AC091563.1*	*AC012123.1*	*RHOV*	*ZNF175*	*SIPA1L3*	*GPR89B*
Fold 5	*LINC01679*	*ICA1L*	*GPR171*	*THPO*	*AC091563.1*	*AC012123.1*	*RHOV*	*ZNF175*	*TINCR*	*ANKS4B*

**Table 9:** LogFC and adjusted *p*-values (calculated by the t-statistic test moderated by an empirical Bayes method and adjusted using the BH method) for the differential expression of the top 12 DEGs, identified with the lowest logFC values, in the two major histological subtypes of cervical cancer using the combined dataset encompassing TCGA-CESC and CGCI-HTMCP-CC samples.

Gene	LogFC	adj.P.Val
*DSG3*	5.6369	$7.52\times 10^{-271}$
*MUC5B*	−5.6182	$2.58\times 10^{-216}$
*MSMO1*	5.6171	$1.30\times 10^{-294}$
*AC026725.1*	5.4261	$6.76\times 10^{-263}$
*ICA1L*	5.3658	$1.88\times 10^{-319}$
*DSC3*	5.1875	$1.45\times 10^{-291}$
*AP1G1*	5.0555	$1.35\times 10^{-298}$
*CCDC89*	4.7556	$3.06\times 10^{-276}$
*SNED1-AS1*	4.6948	$5.27\times 10^{-272}$
*AC244034.3*	−4.6899	$1.05\times 10^{-212}$
*AL360182.1*	4.6675	$6.50\times 10^{-237}$
*FBXL13*	−4.6661	$6.26\times 10^{-215}$

## Data Availability

All data used in this work have been obtained from the GDC Portal, from various projects within the TCGA and CGCI programs, all under public access. The references for the downloaded clinical and sample data are accessible through this link: https://github.com/Almorox/MDPI_Journal_GENES_Uterine_Cancers-Characterization_through_Gene_Expression_Analysis/tree/main/Data_References (accessed on 29 January 2024).

## Supplementary Materials

The following supporting information can be downloaded at: https://www.mdpi.com/article/10.3390/genes15030312/s1, Figure S1: Classification of *CORPUS_TUMOR*, *CERVIX_TUMOR* and *HEALTHY* uterine samples: k-NN test macro F1 score using MRMR as feature selection method. The values are presented as a function of the number of biomarkers used; Figure S2: Classification of *CORPUS_TUMOR*, *CERVIX_TUMOR* and *HEALTHY* uterine samples: sum of the test confusion matrices of the 5-fold cross-validation when using the first 10 MRMR selected miRNAs; Figure S3: Classification of *CORPUS_TUMOR*, *CERVIX_TUMOR* and *HEALTHY* uterine samples: sum of the test confusion matrices of the 5-fold cross-validation when using the two-miRNA signature; Figure S4: Boxplots showing the expression of hsa-mir-21 and hsa-mir-10b miRNAs in each uterine sample class (*CORPUS_TUMOR*, *CERVIX_TUMOR* and *HEALTHY*) using all quality samples; Table S1: Downloaded and filtered samples of each class; Table S2: Classification of *CORPUS_TUMOR*, *CERVIX_TUMOR* and *HEALTHY* uterine samples: top 10 MRMR selected miRNAs for each fold (train set) of the 5-fold cross-validation; Table S3: Classification of *CORPUS_TUMOR*, *CERVIX_TUMOR* and *HEALTHY* uterine samples based on a set of miRNAs associated with the identified gene signature: top 10 MRMR selected miRNAs for each fold (train set) of the 5-fold cross-validation; References [14,33,34,35,40] are cited in the Supplementary Materials.

## Abbreviations

The following abbreviations are used in this manuscript:

kNN	k-nearest neighbors
TCGA	The Cancer Genome Atlas
NCI	National Cancer Institute
NHGRI	National Human Genome Research Institute
GDC	Genomic Data Commons
CGCI	Cancer Genome Characterization Initiative
DEGs	Differentially expressed genes
MRMR	Maximum relevance minimum redundancy
RF	Random forest
DA	Disease association
