# Supplementary material for: Gene Expression Analysis for Uterine Cervix and Corpus Cancer Characterization
Source: Genes (Basel). 2024 Feb 28;15(3):312. doi: 10.3390/genes15030312 (PMC10970626; doi:10.3390/genes15030312)
Supplement: Supplementary file 1 [file genes-15-00312-s001.zip › genes-2877300-supplementary.pdf]

Supplementary Data for

Gene expression analysis for Uterine Cervix and Corpus Cancers Characterization

Lucía Almorox, Laura Antequera, Ignacio Rojas, Luis Javier Herrera and Francisco M. Ortuño

This PDF document includes:

Supplementary text
Figures: S1 to S4
Tables: S1 to S3
References

Supplementary Information Text

Introduction. Given space limitations and the imperative for enhanced clarity and relevance, the materials, methods and results associated with the miRNA-Seq analysis, as introduced in the original manuscript, have been meticulously outlined below.

Materials and Methods

Acquired Data from TCGA Database. Similar to the RNA-Seq data analysis, the data used in this study was acquired through the GDC. In particular, we focused on the primary sites of corpus uteri and cervix uteri, as part of CESC and UCEC TCGA-projects, and the sample types of *primary tumor* and *solid tissue normal.* Subsequently, all BCGSC miRNA Profiling files designated as *miRNA Expression Quantification* data types were downloaded. The corresponding sample sheet and clinical table were also obtained and are accessible in the project's GitHub repository (https://github.com/Almorox/MDPI_Journal_GENES_Uterine_Cancers-Characterization_through_Gene_Expression_Analysis, accessed on 29 January 2024).

KnowSeq. KnowSeq (1) was predominantly employed for the preprocessing and visualization of results in the analysis of miRNA data.

Diana TarBase v8. DIANA-TarBase v8 (https://dianalab.e-ce.uth.gr/html/diana/web/index.php?r=tarbasev8/index, accessed on 8 January 2024) is a reference database devoted to the indexing of experimentally supported miRNA targets. This database is a part of the DIANA (Database for the Integrative Analysis of miRNA) software suite, which includes various tools and databases for the analysis of miRNA-related data. It aims to provide a reliable resource for researchers studying miRNA functions and their roles in gene regulation.

Classification of Healthy, Cervical Cancer and Uterine Corpus Cancer Sample

TCGA Data Preprocessing. After downloading and preprocessing the data, Table S1 displays the number of samples in each class. The number of available variables (i.e., miRNAs) is 1282.

Table S1. Downloaded and filtered samples of each class.

Class	Description	Project	Downloaded	Quality samples
CERVIX_TUMOR	Cervix cancer	TCGA-CESC	309	309
CORPUS_TUMOR	Uterine corpus cancer	TCGA-UCEC	546	544
HEALTHY	Non-cancerous cervix and uterine corpus	TCGA-CESC/UCEC	25	25

5-Fold Cross-Validation Assessment Using MRMR as Feature Selection Method and K-NN as a Supervised Learning Classifier. A 5-fold stratified validation was employed due to the class imbalance. For the case of miRNA, there is no necessity to reduce the number of features since TCGA provides information for all 1282 miRNAs. Building on the results of the prior experiment, MRMR was identified as the most effective method for feature selection, leading to its continued application throughout the remainder of this study. In each fold's training set, a MRMR ranking of 10 miRNAs was obtained and utilized to train and test kNN models (Table S2). For each number of miRNAs (ranging from 1 to 10), the mean training and test F1 macro scores across the five folds were calculated (Figure S1).

Table S2. Classification of *CORPUS_TUMOR*, *CERVIX_TUMOR* and *HEALTHY* uterine samples: top 10 MRMR selected miRNAs for each fold (train set) of the 5-fold cross-validation.

	miRNA 1	miRNA 2	miRNA 3	miRNA 4	miRNA 5	miRNA 6	miRNA 7	miRNA 8	miRNA 9	miRNA 10
**Fold1**	hsa-mir-21	hsa-mir-139	hsa-mir-622	hsa-mir-10b	hsa-mir-4432	hsa-mir-4751	hsa-mir-5589	hsa-mir-3195	hsa-mir-3164	hsa-mir-373
**Fold2**	hsa-mir-21	hsa-mir-139	hsa-mir-4532	hsa-mir-10b	hsa-mir-622	hsa-mir-3164	hsa-mir-4751	hsa-mir-3659	hsa-mir-5580	hsa-mir-3195
**Fold3**	hsa-mir-21	hsa-mir-139	hsa-mir-4432	hsa-mir-10b	hsa-mir-3659	hsa-mir-622	hsa-mir-6805	hsa-mir-5580	hsa-mir-3195	hsa-mir-4751
**Fold4**	hsa-mir-21	hsa-mir-139	hsa-mir-4532	hsa-mir-10b	hsa-mir-5589	hsa-mir-6809	hsa-mir-4751	hsa-mir-3164	hsa-mir-3659	hsa-mir-622
**Fold5**	hsa-mir-21	hsa-mir-4532	hsa-mir-4418	hsa-mir-10b	hsa-mir-373	hsa-mir-622	hsa-mir-3659	hsa-mir-5580	hsa-mir-650	hsa-mir-5589

Table S2 displays the MRMR ranking of each fold from the 5-fold cross-validation. Among the top 2 MRMR miRNAs, only the *hsa-mir-21* and *hsa-mir-10b* combination is observed in the five folds.

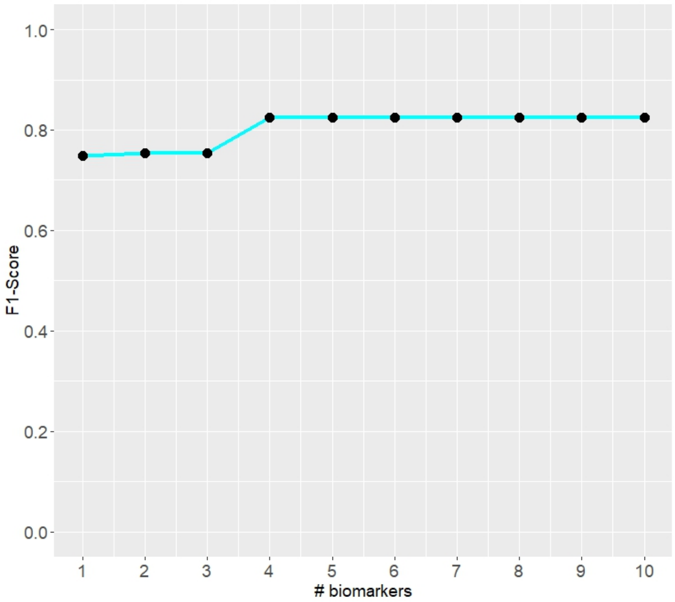

Fig. S1. Classification of *CORPUS_TUMOR*, *CERVIX_TUMOR* and *HEALTHY* uterine samples: k-NN test macro F1 score using MRMR as feature selection method. The values are presented as a function of the number of biomarkers used.

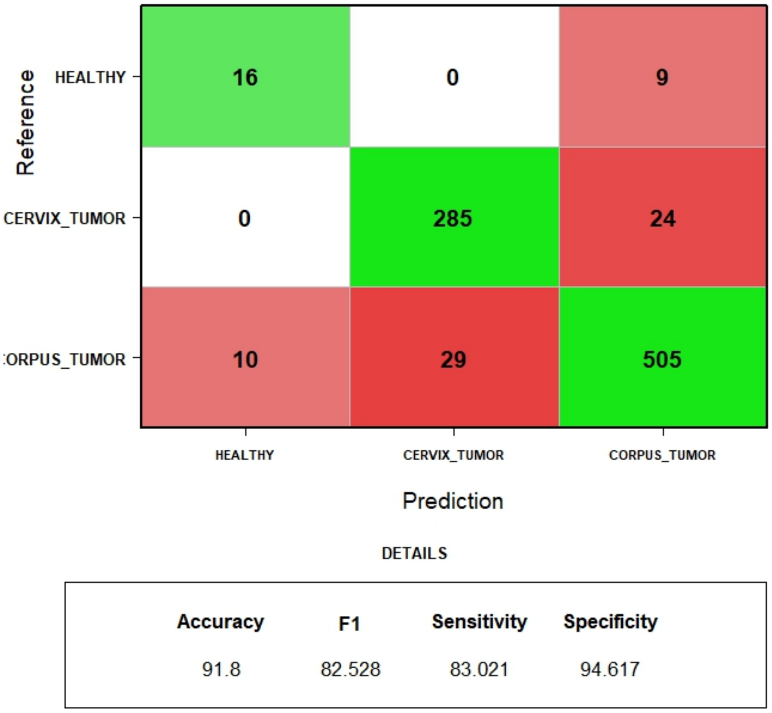

Fig. S2. Classification of *CORPUS_TUMOR*, *CERVIX_TUMOR* and *HEALTHY* uterine samples: sum of the test confusion matrices of the 5-fold cross-validation when using the first 10 MRMR selected miRNAs.

As observed in Figures S1 and S2, better classification performance is achieved when predicting samples from RNA-Seq compared to MiRNA-Seq omics. In Figure S2, it is illustrated that classifying uterine body cancer samples is particularly difficult; distinguishing them from healthy or cervical cancer classes is challenging. Specifically, 10 samples of uterine body cancer were misclassified as healthy samples, and 29 as cervical cancer samples. Similarly, 24 samples of cervical cancer were misclassified as uterine body cancer.

5-Fold Cross-Validation Assessment Using the miRNAs regulating the gene signature considered in the RNA-Seq study. An additional similar analysis was performed. In this case, a reduced set of variables was considered, consisting of the miRNAs that regulate the expression of the genes in the gene signature considered in the RNA-SEQ study (*VWCE - CLDN15 - ADCYAP1R1*). Currently, according to *Diana Tarbase v8*, there is a total of 26 miRNAs regulating the mentioned genes.

Considering a reduced set of miRNAs for feature selection does not enhace the classification performance. Similar accuracy and F1 score values are obtained compared to the analysis that involved all 1282 miRNAs.

Once again, across various training scenarios, *hsa-mir-21* and *hsa-mir-10b* consistently occupy the first and third positions (Table S3). This observation suggests that these variables demonstrate both robust stability and significance in predicting the sample class. Furthermore, it is important to highlight that this analysis strengthens the positive results achieved with RNA-SEQ omics, since these two selected miRNAs are documented in *Diana Tarbase* for regulating the expression of the genes *VWCE* and *ADCYAP1R1*, respectively.

According to our findings, both *hsa-mir-21* and VWCE are overexpressed in cervical cancer compared to the other two sample classes. This observation may seem unexpected, given that miRNAs are traditionally known to suppress the expression of their target mRNAs. However, recent studies have shown that miRNAs, along with their associated protein complexes (microribonucleoproteins or microRNPs), can also play a role in posttranscriptionally stimulating gene expression through direct and indirect mechanisms. The upregulation mediated by microRNAs depends on the specific conditions experienced by different target mRNAs. For instance, a miRNA can either downregulate or upregulate the same mRNA depending on whether the cell is cancerous or not (5). Therefore, further investigation is necessary to confirm whether *hsa-mir-21* upregulates VWCE in cervical cancer cells (similarly, the relationship between *hsa-mir-10b* and ADCYAP1R1 needs clarification). Currently, our knowledge is limited to Tarbase's indication of a relationship between each of these miRNA-gene pairs.

Table S3. Classification of *CORPUS_TUMOR*, *CERVIX_TUMOR* and *HEALTHY* uterine samples based on a set of miRNAs associated with the identified gene signature: top 10 MRMR selected miRNAs for each fold (train set) of the 5-fold cross-validation.

	**miRNA 1**	**miRNA 2**	**miRNA 3**	**miRNA 4**	**miRNA 5**	**miRNA 6**	**miRNA 7**	**miRNA 8**	**miRNA 9**	**miRNA 10**
**Fold1**	hsa-mir-21	hsa-mir-1295a	hsa-mir-10b	hsa-mir-1226	hsa-mir-296	hsa-mir-520f	hsa-mir-2355	hsa-mir-615	hsa-mir-365a	hsa-mir-26b
**Fold2**	hsa-mir-21	hsa-mir-1226	hsa-mir-10b	hsa-mir-296	hsa-mir-520f	hsa-mir-2355	hsa-mir-615	hsa-mir-1295a	hsa-mir-365a	hsa-mir-876
**Fold3**	hsa-mir-21	hsa-mir-1295a	hsa-mir-10b	hsa-mir-876	hsa-mir-296	hsa-mir-520f	hsa-mir-423	hsa-mir-2355	hsa-mir-615	hsa-mir-365a
**Fold4**	hsa-mir-21	hsa-mir-1295a	hsa-mir-10b	hsa-mir-1226	hsa-mir-296	hsa-mir-2355	hsa-mir-27a	hsa-mir-615	hsa-mir-876	hsa-mir-423
**Fold5**	hsa-mir-21	hsa-mir-1295a	hsa-mir-10b	hsa-mir-1226	hsa-mir-296	hsa-mir-520f	hsa-mir-2355	hsa-mir-615	hsa-mir-876	hsa-mir-3619

5-Fold Cross-Validation Assessment Using the two-miRNA signature. Based on the feature selection from the previous analyses, we have again employed a 5-fold stratified validation using only the following miRNAs: hsa-mir-21 and hsa-mir-10b.

In Figure S3, it is observed that the two-miRNA signature is reliably stable to provide nearly the same classification F1-score as the full dataset (Figure S2) and specifically, it outperforms it in terms of accuracy. It suggests that these miRNAs are highly informative and capture essential patterns in the data, while the presence of redundant or irrelevant features in the full dataset may introduce noise and fail to contribute meaningfully to the analysis.

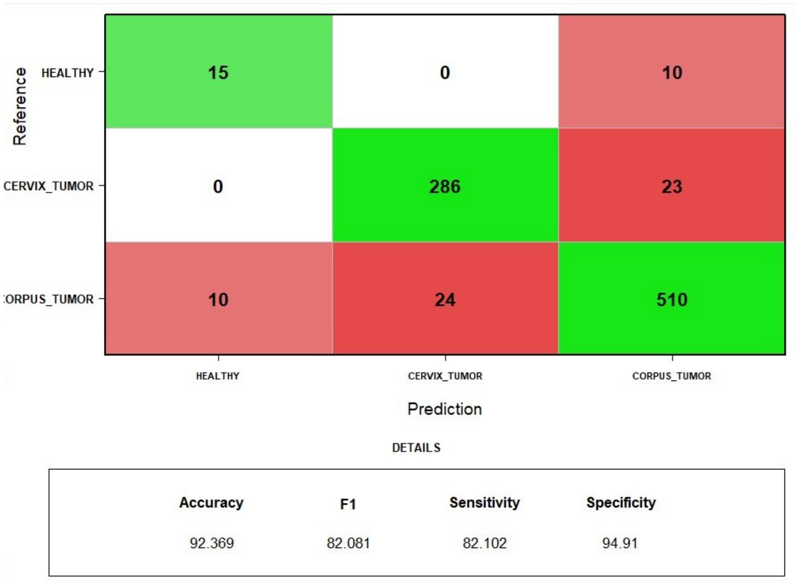

Fig. S3. Classification of *CORPUS_TUMOR*, *CERVIX_TUMOR* and *HEALTHY* uterine samples: sum of the test confusion matrices of the 5-fold cross-validation when using the two-miRNA signature.

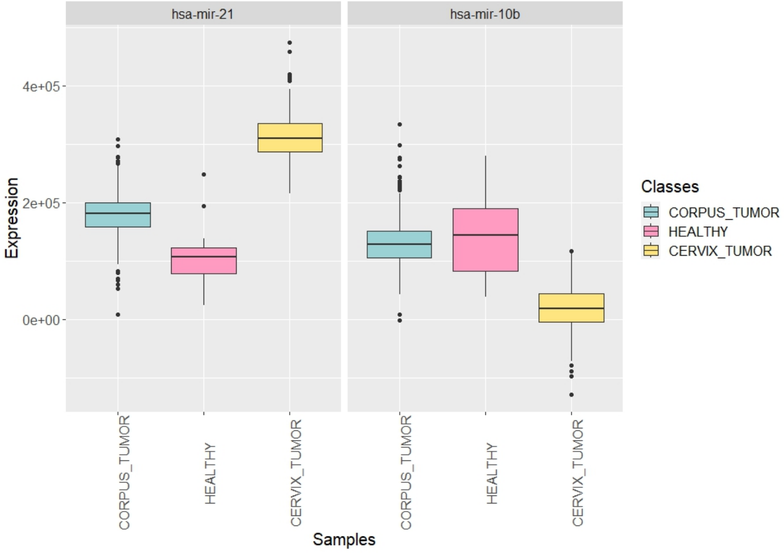

Fig. S4. Boxplots showing the expression of hsa-mir-21 and hsa-mir-10b miRNAs in each uterine sample class (*CORPUS_TUMOR*, *CERVIX_TUMOR* and *HEALTHY*) using all quality samples

MiRNAs Annotation: *hsa-mir-21* and *hsa-mir-10b*. Figure S4 displays boxplots of the expression levels of ***hsa-mir-21*** and ***hsa-mir-10b*** in each sample class. ***hsa-mir-21*** shows overexpression in uterine cervix tissue compared to healthy uterine tissue. Furthermore, this overexpression is more prominent in the cervix than in the corpus, with the distribution of the uterine corpus cancer class being more similar to the healthy class. Conversely, ***hsa-mir-10b*** is underexpressed in cancerous cervical uterine tissue compared to healthy uterine tissue and uterine corpus.

As referenced in (3), ***miR-21*** (***hsa-mir-21*** or ***microRNA-21***) is not only associated with uterine cancer, but it is also highly expressed in a wide variety of cancers and linked to several oncogenic characteristics. In the landscape of miRNAs linked to cancer progression, it emerged as one of the initial oncogenic miRNAs identified. The overexpression of ***miR-21*** has been identified in processes such as cell proliferation, migration, invasion, metastasis, and regulation of apoptosis in cancer.

According to (4), research studies have shown that ***miR-10b*** (***hsa-mir-10b***) can play a role in promoting tumor growth, invasion, and metastasis in different types of cancers. Its dysregulation has been observed in breast cancer, pancreatic cancer

and colorectal cancer. Only one study from 2012 (2) has been identified regarding ***miR-10b*** and cervical cancer metastasis. This study included 44 patients with small cell cervical cancer (SCCC). It was discovered that ***miR-10b*** was downregulated in advanced-stage SCCC tissues compared to early-stage SCCC tissues. However, ***miR-10b*** did not show association with metastasis.

References

1. Daniel Castillo-Secilla, Juan Manuel Gálvez, Francisco Carrillo-Perez, Marta Verona-Almeida, Daniel Redondo-Sánchez, Francisco Manuel Ortuno, Luis Javier Herrera, and Ignacio Rojas. KnowSeq r-bioc package: The automatic smart gene expression tool for retrieving relevant biological knowledge. *Computers in Biology and Medicine*, 133:104387, June 2021.
2. Long Huang, Jia-Xin Lin, Yan-Hong Yu, Mei-Yin Zhang, Hui-Yun Wang, and Min Zheng. Downregulation of six micrornas is associated with advanced stage, lymph node metastasis and poor prognosis in small cell carcinoma of the cervix. *PLoS ONE*, 7(3):e33762, March 2012.
3. Jiho Rhim, Woosun Baek, Yoona Seo, and Jong Heon. Kim. From molecular mechanisms to therapeutics: Understanding microrna-21 in cancer. *Cells*, 11(18):2791, September 2022.
4. Patrick Sheedy and Zdravka Medarova. The fundamental role of mir-10b in metastatic cancer. *Am. J. Cancer Res.*, 8(9):1674–1688, September 2018.
5. Shobha Vasudevan. Posttranscriptional upregulation by micrornas. *WIREs RNA*, 3(3):311–330, November 2011.
